# Extracellular microRNAs exhibit sequence-dependent stability and cellular release kinetics

**DOI:** 10.1080/15476286.2019.1582956

**Published:** 2019-03-05

**Authors:** Anna M. L. Coenen-Stass, Marie J. Pauwels, Britt Hanson, Carla Martin Perez, Mariana Conceição, Matthew J. A. Wood, Imre Mäger, Thomas C. Roberts

**Affiliations:** a Department of Physiology, Anatomy and Genetics, University of Oxford, Oxford, UK; b VIB-UGent Center for Inflammation Research, Department of Biomedical Molecular Biology, Ghent University, Ghent, Belgium; c Department of Biomedical Molecular Biology, Ghent University, Ghent, Belgium; d Department of Paediatrics, University of Oxford, Oxford, UK; e Institute of Technology, University of Tartu, Tartu, Estonia; f Sanford Burnham Prebys Medical Discovery Institute, Development, Aging and Regeneration Program, La Jolla, CA, USA

**Keywords:** Extracellular microRNA, ex-miRNA, kinetics, serum, half-life, microRNA, miRNA

## Abstract

Multiple studies have described extracellular microRNAs (ex-miRNAs) as being remarkably stable despite the hostile extracellular environment, when stored at 4ºC or lower. Here we show that many ex-miRNAs are rapidly degraded when incubated at 37ºC in the presence of serum (thereby simulating physiologically relevant conditions). Stability varied widely between miRNAs, with half-lives ranging from ~1.5 hours to more than 13 hours. Notably, ex-miRNA half-lives calculated in two different biofluids (murine serum and C2C12 mouse myotube conditioned medium) were highly similar, suggesting that intrinsic sequence properties are a determining factor in miRNA stability. By contrast, ex-miRNAs associated with extracellular vesicles (isolated by size exclusion chromatography) were highly stable. The release of ex-miRNAs from C2C12 myotubes was measured over time, and mathematical modelling revealed miRNA-specific release kinetics. While some ex-miRNAs reached the steady state in cell culture medium within 24 hours, the extracellular level of miR-16 did not reach equilibrium, even after 3 days in culture. These findings are indicative of miRNA-specific release and degradation kinetics with implications for the utility of ex-miRNAs as biomarkers, and for the potential of ex-miRNAs to transfer gene regulatory information between cells.

## Introduction

Extracellular microRNAs (ex-miRNAs) are present in a wide variety of biofluids, including serum, plasma, urine, cerebral spinal fluid, saliva, tears, milk, and seminal fluid [1]. As such, ex-miRNAs have been investigated as minimally-invasive biomarkers for a range of physiological and pathophysiological states including tissue damage [2], cancer [3–5], infectious disease [6], atherosclerosis [7], pregnancy [8], and diabetes [9]. In particular, our group and others have investigated the utility of ex-miRNAs in the context of the progressive muscle-wasting disorder Duchenne muscular dystrophy (DMD) [10–17]. A set of highly muscle-enriched miRNAs, the classical myomiRs (consisting of the miR-1/206 family, MIPF0000038, and miR-133 family, MIPF0000029 [18]) are elevated in the serum of DMD patients and dystrophic animal models [10–17]. These myomiRs are known to regulate myoblast proliferation and differentiation by suppressing expression of stemness factors such as PAX3 and PAX7, inhibiting DNA synthesis via POLA1, and promoting MEF2 expression via the repression of HDAC4 [19–22]. miR-206 in particular has been shown to play a key role in muscle regeneration *in vivo* [23,24]. MyomiR release from dystrophic muscle is associated with both degenerative [11,15] and regenerative [12–14,25] processes. Whether or not these ex-miRNAs can act to transfer gene regulatory information between cells remains an exciting area of ongoing research [26–28].

The concentration of miRNAs in the extracellular space is necessarily a function of the rates of export, re-uptake, and degradation. Several groups have commented on the remarkable stability of ex-miRNAs at room temperature [3,29], in frozen storage [30,31], and following multiple freeze-thaw cycles [3,32,33]. Given that unmodified naked RNA oligonucleotides are rapidly degraded (on the order of seconds) by extracellular RNase activity if added directly to serum, or after intravenous injection [3,34], the stability of ex-miRNAs was somewhat surprising. The discovery of ex-miRNAs in biofluids coincided with an explosion of interest in extracellular vesicles (EVs) as both mediators of cell-to-cell signaling [35–40], and as potential therapeutic delivery vehicles [41]. Indeed, encapsulation of miRNAs within lipid vesicles (i.e. exosomes, microvesicles, and apoptotic bodies) would provide a simple and elegant explanation for ex-miRNA stability in the hostile environment of the extracellular space [42]. However, while miRNAs are certainly present within EVs [39], the vast majority of ex-miRNA have been shown to be non-vesicular [43–47]. Consistent with these findings, we observed that ~99% of extracellular miR-1, miR-133a and miR-206 were found to be non-vesicular in dystrophic serum as determined by multiple orthogonal methodologies [13,14]. Furthermore, these miRNAs were found to immunoprecipitate with AGO2 and APOA1, indicating that they are likely stabilized in extracellular-ribonucleoprotein (ex-RNP) complexes [13].

Given that multiple groups have proposed that ex-miRNAs can act as cell-to-cell signaling molecules, their stability under physiological conditions is an unaddressed issue of high importance. Here we have investigated the nuclease stability of ex-miRNAs at 37°C in murine serum and cell culture conditioned medium. We further utilized myotube cell cultures to model ex-miRNA release kinetics. From these data we have generated a mathematical model which describes the behaviour of this system, and calculated decay/release rates for each ex-miRNA tested. These findings have important implications for the use of ex-miRNAs as biomarkers, and for their functional relevance.

## Results

### Extracellular miRNAs in cell culture-conditioned medium are degraded at different rates under physiological conditions

To investigate the stability of ex-miRNAs, we sought to determine the half-lives of myomiRs (miR-1, miR-133a and miR-206) and non-myomiR controls (miR-16 and let-7a) at physiological temperature (i.e. 37°C) in conditioned medium taken from C2C12 myotube cultures. C2C12 cells are a mouse myoblast cell line that differentiates into multinucleate myotubes in low serum conditions (i.e. 2% horse serum, HS). C2C12 cells were cultured in differentiation conditions for 6 days, so as to reach a stage at which ex-myomiRs were being secreted into the cell culture medium at high levels [14]. Cell culture medium was then collected (72 hours after the previous change of the medium) and incubated at 37°C to simulate human/mouse body temperature. Aliquots of conditioned medium were removed over a series of time points (i.e. 0, 1.5, 3, 6, 10, 24, 48, and 76 hours) and miRNA abundance determined by RT-qPCR. Curves were fit to experimental data by non-linear regression using a one phase exponential decay model. This experimental design allows for the degradation rate to be measured in the absence of further miRNA export or re-uptake.

**Figure 1.**
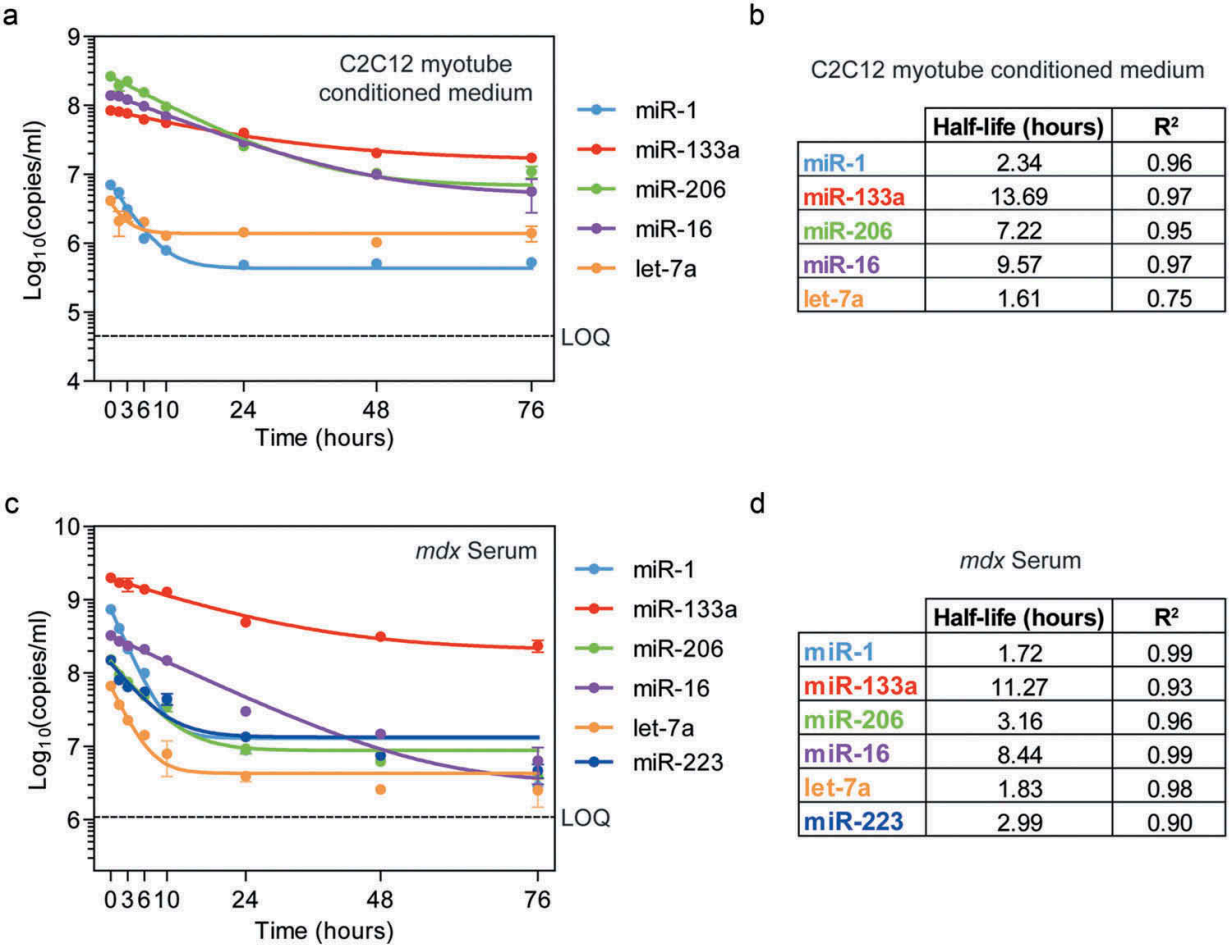
Extracellular miRNA stability at physiological temperature. (a) C2C12 myotube conditioned medium was incubated at 37ºC and miRNA abundance determined at each time point by RT-qPCR. (b) Table of miRNA half-lives and goodness-of-fit (R^2^) values for each miRNA in C2C12 myotube conditioned medium. (c) Serum from dystrophic *mdx* mice was incubated at 37ºC as indicated and miRNA abundance determined at each time point. (d) Table of miRNA half-lives and goodness-of-fit (R^2^) values for each miRNA in dystrophic serum. Curves were fitted by non-linear regression using a one phase exponential decay model. Values are mean±SEM, *n* = 2. LOQ, limit of quantification.

In contrast with previous reports investigating ex-miRNA stability in frozen storage or at room temperature [3,29,31], we observed a progressive decline in all ex-miRNA species analyzed after incubation under simulated physiological conditions (Figure 1(a)). Strikingly, the ex-miRNAs exhibited a wide range of stabilities. For example, miR-1 and let-7a were rapidly degraded during the first 10 hours of incubation, as reflected in their short half-lives (2.3 and 1.6 hours respectively). In contrast, miR-133a was relatively stable over the course of the experiment, with a calculated half-life of >13 hours. miR-206 and miR-16 exhibited intermediate half-lives (7.2 and 9.6 hours respectively) (Figure 1(b)). All ex-miRNAs reached plateaus at a level 2–3 logs above the limit of quantitation (LOQ) for each assay, indicating the presence of a nuclease-resistant fraction for each measured ex-miRNA.

### Extracellular miRNAs in dystrophic serum are degraded at different rates under physiological conditions

To determine whether the findings from the C2C12 myotube cultures could be extended to the *in vivo* situation, we performed an analogous experiment using serum samples collected from dystrophin-deficient *mdx* mice (the most commonly used mouse model of DMD). *mdx* mice undergo a period of muscle degeneration and regeneration which is reminiscent of the pathology observed in the muscles of DMD patients [48]. Notably, myomiR levels are highly elevated (50–100-fold relative to wild-type controls) in the serum of *mdx* mice [10–12], and so *mdx* serum provides a useful model in which to investigate ex-miRNA stability.

Frozen serum samples were thawed on ice, placed in a 37°C incubator, and aliquots collected over a series of time points (i.e. 0, 1.5, 3, 6, 10, 24, 48, and 76 hours). RNA was extracted and miRNA levels determined as described above. An additional non-myomiR control miRNA, miR-223, was included in the analysis. This granulocyte-specific miRNA has previously been used as an endogenous reference control for data normalization by our group [12,49] and others [11,50]. Note that miR-223 is not expressed in C2C12 myotubes and was therefore not analyzed in the cell culture studies described above.

Remarkably, the patterns of miRNA degradation in *mdx* serum were similar to those observed in C2C12 myotube conditioned medium (Figure 1(c)). miR-1 was degraded at the most rapid rate, decreasing by ~10-fold within the first 10 hours. miR-206 and miR-233 had intermediate half-life values (~3 hours), and miR-133a and miR-16 degraded more slowly (>11 and >8 hour half-lives respectively) (Figure 1(d)). As with the results for C2C12 myotube conditioned medium, levels of each ex-miRNA reached a plateau at a level 1–3 logs over the assay LOQ values.

miRNA half-lives were highly correlated between *mdx* serum and C2C12 myotubes conditioned supernatant (Pearson *r *= 0.948, *P *= 0.014) indicative of similar degradation kinetics observed in biofluids of very different compositions (Figure S1).

### Extracellular miRNA stability is correlated with sequence GC content

The observation that different miRNAs exhibit distinct stability kinetics is surprising given that these miRNAs are assumed to be protected from nuclease degradation in a similar manner (i.e. through protein/lipoprotein complex formation). We therefore reasoned that their stabilities might be determined by the intrinsic properties of the miRNA sequence itself. Notably, the miR-1 family miRNAs (miR-1 and miR-206) exhibited quite different decay kinetics despite being closely-related. Inspection of the sequences for these miRNAs revealed differences at four nucleotide positions; one uridine and three adenosine base positions in miR-1 are occupied by guanosine bases at the equivalent positions in miR-206 (Figure S2(a)). We therefore hypothesised that the stability of a given ex-miRNA might be associated with its GC content. Indeed, ex-miRNA half-life and GC content were positively correlated (Pearson *r *= 0.918, *P *= 0.0277 for C2C12 myotube conditioned medium and Pearson *r *= 0.824, *P *= 0.0438 for *mdx* serum) (Figure S2(b,c)).

### Extracellular vesicle-associated miRNAs are stable

In the experiments described above, we observed that ex-miRNA levels reached a plateau after incubation at 37°C which was much higher than the calculated assay LOQs. These findings suggest the existence of a sub-population (comprising between 4% and 34% of the starting material) of nuclease-resistant ex-miRNAs that remain stable, even after incubation at 37°C for 3 days. One possibility is that such miRNAs might reside within EVs which provide protection from nuclease-mediated degradation [51]. We therefore sought to assess miRNA stability in EVs incubated at 37°C.

**Figure 2.**
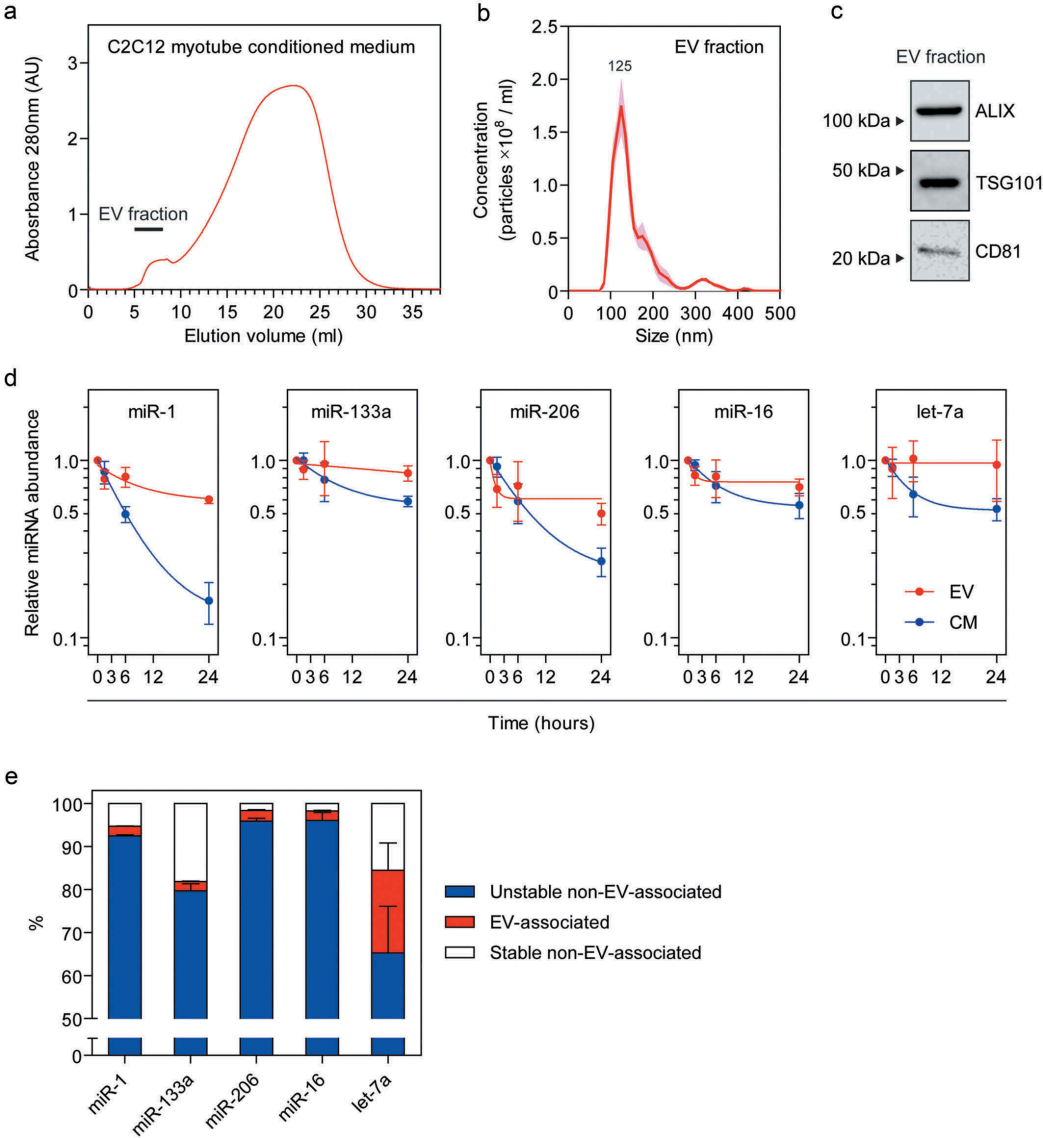
Stability of extracellular vesicle-associated miRNAs at physiological temperature. Extracellular vesicles (EVs) were isolated from C2C12 myotube conditioned medium (CM) by tangential flow filtration and size exclusion liquid chromatography. (a) 280 nm absorbance trace for liquid chromatography fractions. The pooled fractions which contain the EVs are indicated on the plot. Isolated EVs were analyzed by (b) nanoparticle tracking analysis, and (c) Western blot for exosome markers (ALIX, TSG101, and CD81). (d) Isolated EV or unfractionated CM samples were incubated at 37ºC and miRNA abundance determined at each time point by RT-qPCR. (e) Estimation of the proportion of ex-miRNAs that are unstable and non-vesicular (i.e. the amount of miRNA that is degraded after incubation at 37°C for 76 hours, from Figure 1), EV-associated (i.e. the proportion of ex-miRNA contained in the EV fractions after liquid chromatography), and the stable non-vesicular (i.e. the proportion of ex-miRNA remaining after incubation at 37°C for 76 hours minus the EV-associated fraction). Values are mean±SEM, *n *= 3.

EVs were isolated from C2C12 myotube conditioned medium using a combination of ultra-filtration (using tangential flow) and size exclusion liquid chromatography (UF-LC), a methodology that we have previously developed in order to generate concentrated, pure, and intact vesicles devoid of ex-RNP contamination [52]. LC performance was monitored by UV spectroscopy at 280 nm (Figure 2(a)). The high molecular weight eluates (containing EVs) were collected, pooled, and characterized. Nanoparticle tracking analysis showed a well-defined peak with a modal size of 125 nm, consistent with the expected size range for exosome-like EVs (Figure 2(b)). Similarly, the known exosome markers; ALIX, TSG101, and CD81 were readily detectable in the pooled EV fractions (Figure 2(c)).

During EV purification by UF-LC, soluble extracellular proteins (including RNAses) are separated from the vesicles. Given that degradation of ex-miRNAs is mediated by extracellular RNases [51], direct comparison of miRNA stability in conditioned medium with purified EVs is potentially misleading. We therefore sought to reconstitute the hostile RNase-rich extracellular environment by resuspending purified C2C12 myotube-derived EVs in PBS containing 2% HS in order to mimic the biological composition of the neat conditioned medium.

Concentrated EV preparations, and unfractionated conditioned medium from the parent cultures, were placed at 37°C and aliquots collected after 0, 1.5, 6, and 24 hours incubation. RNA was extracted and miRNA abundance levels determined by RT-qPCR. Three independent C2C12 myotube cultures and EV isolations were performed on separate days for this analysis. Results for conditioned medium samples were similar to those reported above (Figure 1(a)), whereby a progressive decrease in miRNA levels was observed in all cases (Figure 2(d)). Furthermore, the rate of decline was different between miRNAs, with miR-1 and let-7a exhibiting rapid decreases, while miR-133a and miR-16 had slower rates of decline (Figure 2(d)). In contrast, miRNA levels in the EV fractions were relatively stable. Initial small, but rapid, decreases were observed in the first 1.5 hours for miR-1, miR-206 and miR-16, which may represent miRNAs that are associated with the outside surface of the EVs, or with the carryover of vesicle-free ex-miRNA during EV purification, and therefore not protected from extracellular RNase activity (Figure 2(d)). EV-associated miR-206, miR-16 and let-7a reached early plateaus. Indeed, EV-associated let-7a showed no reduction below the initial starting amount. The proportions of ex-miRNAs that were contained in EVs were calculated to be ~2% for all miRNAs except for let-7a, which was enriched in EVs to a greater extent (~10–30%) (Figure 2(e)). These values are similar to our previous estimates for these miRNAs [13,14]. Notably, let-7a has been previously reported to be preferentially enriched in EVs derived from human plasma [44], which may account for the discrepancy between this miRNA and the others measured here. These data suggest that ex-miRNAs contained within EVs are much more stable than non-vesicular ex-miRNAs. However, the observation that EV-associated miRNAs constitute the minority of the total ex-miRNA pool means that these findings cannot fully account for the nuclease-resistant fraction observed in the plateau phase in Figure 1. These findings therefore imply the existence of a further, non-vesicular subpopulation of ex-miRNAs that is highly resistant to degradation at 37°C (Figure 2(e)).

It has recently been suggested that ex-miRNAs present in cell culture media supplements such as fetal bovine serum (FBS) and HS can confound cell culture investigations of extracellular RNA function [53]. All miRNAs investigated in this study are completely conserved between human, mouse, cow and horse, with the exception of miR-16, which exhibits some minor differences at the 3ʹ terminus (Figure S3(a)). As such, the murine miRNA TaqMan assays utilized here are expected to also amplify homologous miRNAs from cow and horse sera. To exclude the possibility that detection of non-murine miRNAs is complicating our analysis, we extracted RNA from undiluted HS and FBS and measured miRNA levels by RT-qPCR. Importantly, these miRNAs were generally detected at very low levels in HS (i.e. miR-1 was mostly undetected, miR-133a Cq>36, miR-206 Cq>36, let-7a Cq>34, and miR-16 Cq ~30) (Figure S3(b)). The very low levels of the miRNAs-of-interest in these undiluted supplements, and the fact that they were further diluted in the cell culture medium by 6.67-fold and 50-fold for FBS (for growth medium) and HS (for differentiation medium) respectively, suggests that exogenous serum contaminants are unlikely to have significantly affected the findings reported in this study. Similarly, EVs isolated from an equivalent volume of HS exhibited exosome-like particle sizes, but were not enriched for murine exosome markers, and contained ex-myomiRs at levels ~200-fold lower than in C2C12 myotube-derived EVs (data not shown).

### Release of extracellular miRNA from myotube cultures

**Figure 3.**
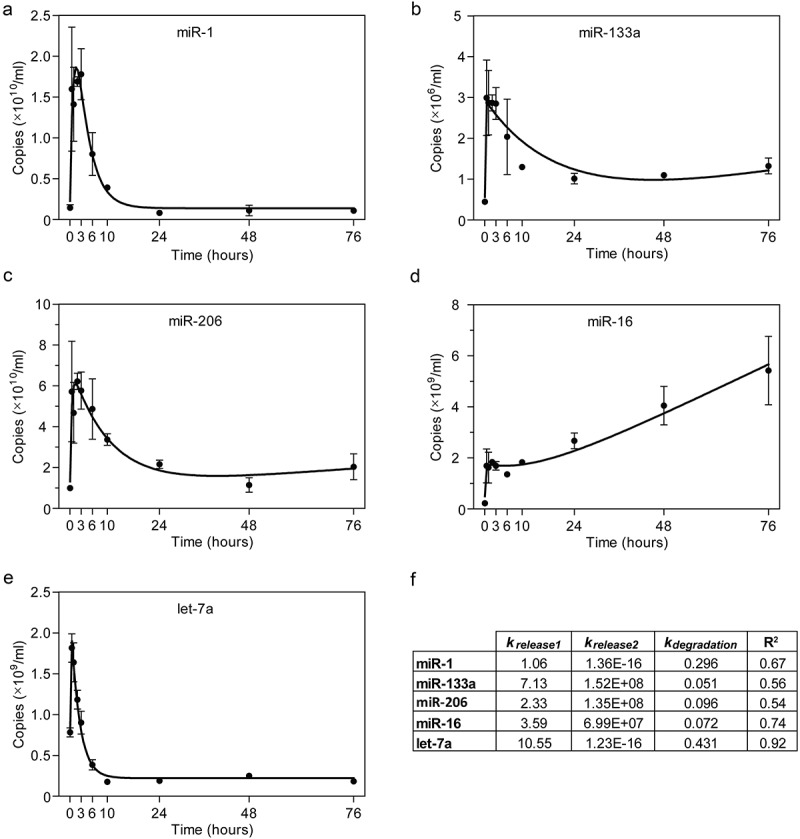
Analysis of extracellular miRNA release after cell culture medium change. ex-miRNA release was modelled using C2C12 myotube cultures. ex-miRNA levels were determined by RT-qPCR in C2C12 myotube conditioned medium at the indicated time points after changing the medium at time zero for (a) miR-1, (b) miR-133a, (c) miR-206, (d) miR-16, and (e) let-7a. Values are mean±SEM, *n *= 3. (f) Table of kinetic constants and model goodness-of-fit (R^2^) values for each miRNA.

Having found that secreted ex-miRNAs exhibit sequence-dependent decay kinetics, we were further motivated to examine whether similar differences are observed in the rate of ex-miRNA cellular release. To investigate the ex-miRNA release kinetics in cell culture, C2C12 cells were first differentiated to form myotubes. The culture medium was discarded, the cells were washed with PBS in order to remove any residual ex-miRNAs that may be bound to cells or the cell culture dish, and then fresh differentiation medium was added. Subsequently, conditioned medium was harvested at various time points (0, 0.5, 1, 2, 3, 6, 10, 24, 48, and 72 hours) and ex-miRNA abundance determined by RT-qPCR for miR-1, miR-133a, miR-206, miR-16 and let-7a (Figure 3). This experiment was designed to measure the changes in ex-miRNA levels in cell culture supernatant which are a function of both release and degradation.

Extracellular miRNA concentration was modelled using non-linear regression as a function of three processes; a rapid release (Release1, 1^st^ order), degradation (1^st^ order), and a gradual release (Release2, 0^th^ order). The rate constant for miRNA decay (*k_degradation_*) was determined empirically from the experiments described above (Figure 1). The model is described by Equation (1), where *t* is time and *c* is a constant.

(1) $$[miRNA]=\frac{k_{release1}.[initial]}{k_{degradation}-k_{release1}}.(e^{\left(-k_{release1}.t\right)}-{e^{-k_{{\;}_{degradation}}}}^{.t})+k_{release2}.t.c$$

cdegradation process formedium, the C2C12 myotubes rapidly released miRNAs. The concentration of ex-miRNAs initially peaked between 0.5 and 1 hours (Figure 3(a-e)). The ex-miRNA levels subsequently dropped as a consequence of the degradation process for all miRNAs, except for miR-16. A second, more gradual release phase was observed for miR-133a and miR-206 (Figure 3(b,c)). For the miRNAs with the fastest rates of degradation (miR-1 and let-7a) the Release2 component was negligible, likely as a consequence of parity between the release and degradation processes (Figure 3(a,e)). For these miRNAs, the extracellular concentration remained stable after ~10 hours. In contrast with the other miRNAs, the extracellular concentration of miR-16 increased above the levels observed during the initial rapid release phase (Figure 3(d)). Rate constants for the two release processes were inferred using a combination of experimental data and modelling (Figure 3(f)).

**Figure 4.**
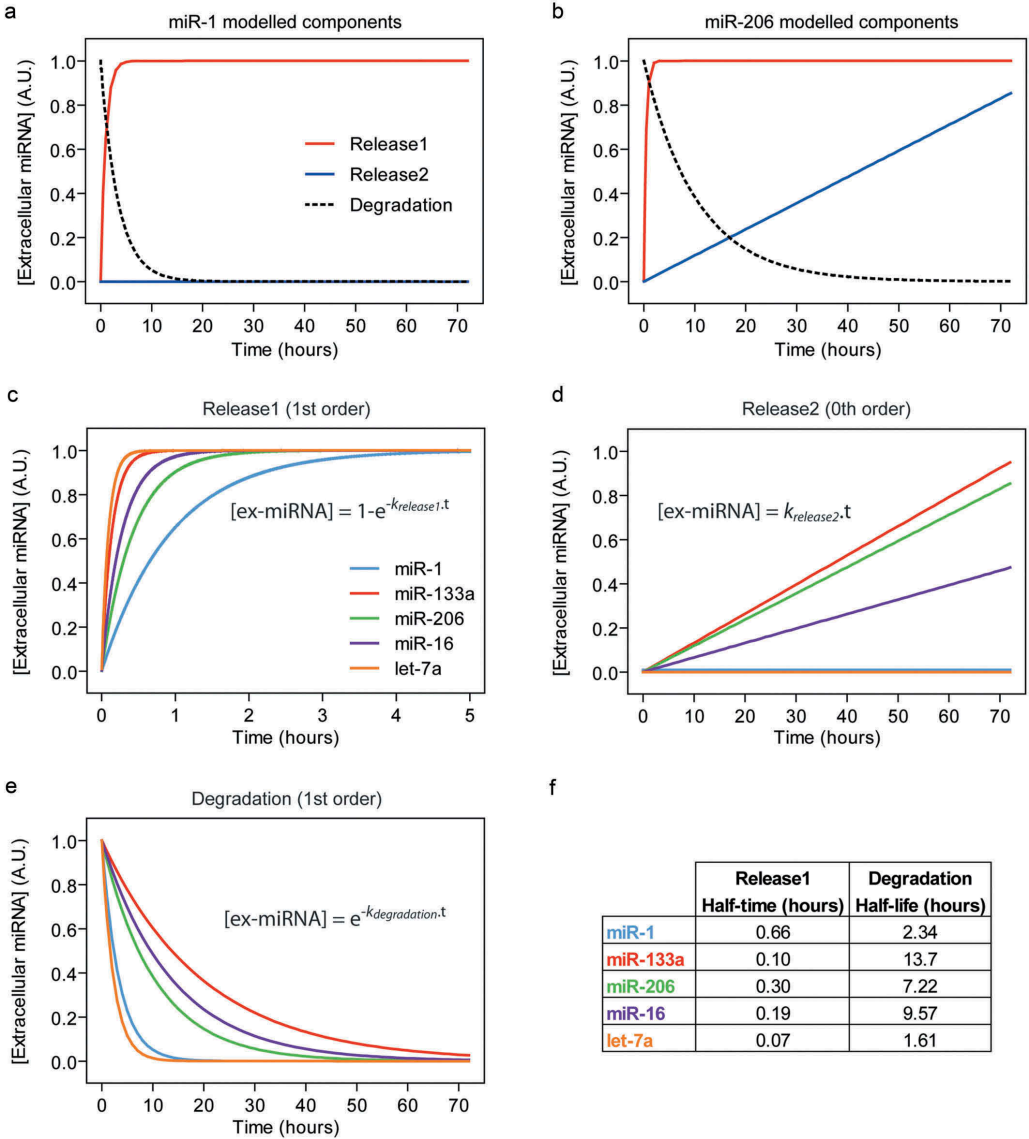
Modelling of extracellular miRNA release kinetics. ex-miRNA release and degradation components were simulated based on a mathematical model fitted to empirical data. The Degradation rate constants were determined using data from Figure 1. The Release1 and Release2 rate constants were determined using data from Figure 3. The three processes contributing to steady-state ex-miRNA levels were plotted separately for two representative miRNAs; (a) miR-1, which exhibits a negligible Release2 component, and (b) miR-206, which exhibits a prominent Release2 component. Simulated data for the three processes contributing to ex-miRNA levels were modelled individually in order to compare miRNAs for (c) the first release process (Release1), (d) the second release process (Release2), and (e) Degradation. Equations used to generate the curves are shown on the plots.

The release and degradation processes were simulated using the model described in Equation (1) and rate constants calculated above. Simulated data for two representative miRNAs (miR-1 and miR-206) illustrate the differences between these three processes (Figure 4(a,b)).

The simulated data for each miRNA is shown whereby each component is plotted separately for Release1 (Figure 4(c)), Release2 (Figure 4(d)) and Degradation (Figure 4(e)). For the Release1 component, kinetics differed between miRNAs, although the half-times were rapid (ranging from 0.07 to 0.7 hours, Figure 4(f)). Interestingly, miR-1 exhibited the longest Relase1 half-time, although there was no significant correlation between ex-miRNA Release1 half-times and GC content in this case (Figure 4(f)). In contrast, the Degradation and Release2 components were strikingly different between miRNAs (Figure 4(d,e)).

## Discussion

Here we have analyzed the stability and release kinetics of ex-miRNAs at physiological temperature, in order to model the likely behaviour of these molecules in biofluids *in vivo*. While ex-miRNAs have been previously described as highly stable at room temperature or when frozen, we observed that miRNAs are progressively degraded when subjected to physiological temperature. Surprisingly, miRNAs were found to exhibit a wide range of stabilities, with half-lives in the range of hours, and remarkably similar results were observed in both myotube-conditioned cell culture medium and murine serum. The investigation of ex-miRNA stability is important for understanding absolute miRNA levels in biofluids. For example, miR-133a was the most highly expressed myomiR in adult *mdx* serum (Figure 1(c)). Given the low stability of miR-1 compared to miR-133a, one might hypothesise that miR-1 could be exported at similar rates as miR-133a *in vivo*, but persists in the circulation for a shorter amount of time. Similarly, differential ex-miRNA stability may account for some of the discrepancies observed between intracellular and extracellular miRNA levels [43]. Differences in ex-miRNA stability may result in a ‘decoupling’ of the relationship between miRNA tissue expression and biofluid abundance which, when measured at the steady state, might erroneously be considered evidence for selective secretion.

Previous investigations of ex-miRNA stability at room temperature have reached different conclusions, with some studies reporting that ex-miRNAs are relatively stable [3,29,33], whereas a study by Köberle et al. reported the opposite [51]. The latter study is notable as several of its findings bear a striking resemblance to those reported here. Specifically, that miRNAs are not stable, and that they are degraded at different rates. Importantly, this study also observed that miR-1 was the least stable, and that miR-16 decayed much more slowly [51], consistent with our findings. The differences between these studies may be accounted for by the limited number of miRNAs tested, the use of human plasma [3], or the relatively short time period analyzed [33].

Notably, a study by Yang *et al*., showed that the small RNA MIR2911 (initially described as a plant miRNA but later shown to be a 26S ribosomal RNA fragment [54]) that is highly stable in serum, is non-exosomal, and GC-rich [55]. While these observations resemble some of those reported in the present study, this small RNA may constitute a unique case. For example, in contrast with the myomiRs studied here [13], serum MIR2911 is insensitive to proteinase K treatment [55].

In contrast with myotube conditioned medium, we observed that ex-miRNAs in isolated EVs were highly stable (Figure 2). However, the majority of studies have shown that ex-miRNAs in conditioned medium are predominantly non-vesicular, suggesting that protection from RNase degradation is primarily mediated by association with ex-RNPs (e.g. Argonaute proteins such as AGO2, HDL components such as APOA1, or other proteins such as NMP1 [13,43,44,47]). It is likely that the various miRNA-binding proteins may provide different degrees of RNase protection.

ex-miRNA half-lives were correlated with GC content, such that the most GC-rich miRNA investigated (miR-133a) was also the most stable. Such a sequence-dependent relationship is surprising, as it would suggest that Watson-Crick base pairing might be involved in stabilizing a particular miRNA. However, many RNA binding proteins interact with RNA substrates in a sequence-independent manner. For example, AGO2 primarily makes contact to the miRNA guide through hydrogen bonds and salt bridges to the phosphodiester backbone, and van der Waals interactions with the ribose sugars [56]. Such binding therefore leaves the Watson-Crick face solvent exposed, such that it is available for binding to a cognate target transcript. *In vitro* studies of recombinant AGO2 suggest that miRNAs are held tightly within the complex [57], and that binding to synthetic complementary target molecules facilitates miRNA unloading [57]. Furthermore, AGO2 exhibits a weaker binding affinity for double-stranded RNA than for single-stranded RNA [58]. It is therefore possible that binding to extracellular AGO2 accounts for the population of ex-miRNAs that are resistant to nuclease degradation, even after incubation at 37°C for 3 days. As such, the ex-miRNAs of the unstable population, which are rapidly degraded at sequence-dependent rates, are likely to reside within distinct ex-RNP complexes.

To date, relatively few studies have investigated the stability of ex-miRNAs, and the majority of these have analyzed RNA stability either at room temperature, or in frozen storage. These studies are important for determining how best to store and handle biofluid samples for analysis of extracellular nucleic acids and/or EVs, and are relevant to the potential utility of miRNA biomarkers. The relative stability (or lack thereof) of ex-miRNAs at physiological temperature is an important factor in terms of their potential for biological functionality. Rapid degradation may limit the capacity of cell-to-cell communication over long distances. Instead, if RNP-associated ex-miRNAs act as signaling molecules at all, it will likely be in a ‘short range’, paracrine manner via the interstitial space.

Several studies have reported EV-mediated miRNA transfer between cells in the muscle niche [26–28,59], although the use of sub-optimal methods of EV isolation (specifically, polymer precipitation using commercial kits [60,61]) in some of these studies limits their usefulness. Importantly, vesicle-associated miRNAs constitute a tiny minority of the total ex-miRNA pool, raising the obvious question as to what the purpose (if any) of the remaining non-vesicular majority is. Indeed, non-vesicular ex-miRNAs have been dismissed as non-functional, cellular waste by some groups [42]. However, asymmetric patterns of intra- and extracellular miRNA levels are potentially indicative of a selective export mechanism, and therefore a regulated process, at least in the case of some miRNAs [43]. To date, there has been no clear demonstration that ex-miRNA-RNP complexes are capable of being taken up by recipient cells in order to induce gene silencing events [62]. Instead, ex-miRNA-RNP complexes may execute a function distinct from their well-established roles in gene regulation.

All ex-miRNAs measured were rapidly released by differentiated C2C12 myotubes within 30 minutes of replacing the culture medium (Figure 3). These findings are similar to those reported by Wang *et al*., whereby a sudden burst of miRNA release was observed in human cancer cells immediately after serum withdrawal [43]. The authors hypothesised that acute serum withdrawal provides a stimulus for miRNA export [43]. However, in the results presented here, the serum concentration was the same both before and after changing the medium, suggesting that the medium change alone (and not necessarily serum withdrawal) is sufficient to induce a similar rapid miRNA release response to that reported by Wang *et al*., Notably, the ex-miRNA levels measured immediately after changing the medium were much higher than background, suggestive of a rapid, and active export mechanism [43]. Alternatively, some miRNA carrier complexes might be attached to the outside of the cell by weak interactions and are subsequently liberated upon the addition of new medium. The requirement for medium change is a potential limitation of the current study as this may constitute an artificial stress situation that is not representative of *in vivo* miRNA release. Methodological improvements that allow for the biochemical tracking of newly synthesised and exported miRNAs without the need to change the culture medium will be required to address this issue.

For non-linear regression of cellular release data (Figure 4), *k_degradation_* was constrained using experimentally determined values from Figure 1(b). However, slightly better goodness-of-fit (R^2^) values could be obtained by not constraining this rate constant, suggesting that secreted factors may influence the rate of ex-miRNA degradation (which are absent in the experiments performed in serum or isolated myotube conditioned medium, Figure 1). However, the measurement of ex-miRNA is inherently prone to noise [32]. Such variation may result in minor differences between experiments which affect the R^2^ value for each ex-miRNA model. Alternatively, there may be some re-uptake of secreted miRNAs that manifests as a ‘degradation-like process’ and which we did not take into account in our model. Even so, the high R^2^ value for let-7a (0.92) suggests that the model is sufficient to explain the majority of the experimental data without the need for an additional re-uptake term in this case.

In conclusion, our findings underline the importance of considering degradation and release kinetics for understanding steady-state ex-miRNA levels. A limitation of our study is the relatively small number of miRNAs investigated (six for conditioned medium and seven for murine serum). Future work must focus on determining the universality of the results presented here, and investigate the mechanisms underlying these observations. Taken together, these findings suggest that changes in sample collection protocol can significantly influence ex-miRNA levels, even prior to sample processing and storage. This must be taken into consideration when working with ex-miRNA based biomarkers. The relative stability of ex-miRNAs also has implications for their capacity to transfer gene regulatory information between cells.

## Materials and methods

### Animal samples

Animal studies were conducted in accordance to procedures approved by the UK home office (project licence 30/2907). 24 week old female dystrophic *mdx* mice (C57BL/10ScSn-*Dmd^mdx^*/J) were sacrificed by escalating CO_2_ concentration and blood was collected immediately *post mortem* from the jugular vein using Microvette CB300 capillary serum collection tubes (Sarstedt, Leicester, UK). Samples were incubated on ice for 1 hour to allow samples to clot and then centrifuged at 10,000 *g* for 5 minutes to pellet cellular blood. The supernatant was carefully removed and transferred to fresh tubes and stored at −80°C. No samples were visibly hemolyzed.

### Cell culture

C2C12 cells were maintained in growth medium (GM; Dulbecco’s Modified Eagle’s Medium (DMEM) supplemented with 15% FBS) and 1% antibiotics/antimycotics (all Life Technologies) at 37°C with 5% CO_2_ and switched to differentiation medium (DM; DMEM supplemented with 2% HS) for 6 days in order to induce the formation of multinucleate myotubes. Cell culture-conditioned medium was centrifuged at 2,000 *g* for 5 minutes at 4°C, and then passed through a 0.22 µm filter to ensure the removal of any residual cells or debris that may have been carried over. Filtered conditioned medium was stored at −80°C.

### miRNA RT-qPCR

Cell culture conditioned medium or serum was incubated at 37°C to simulate physiological temperature and aliquots (25 µl for serum or 300 µl for cell culture conditioned medium) removed at time points as indicated. Liquid samples were immediately placed in TRIzol LS (Thermo Fisher Scientific) in order to prevent further RNA degradation. An external spike-in control oligonucleotide (3 µl of 5 nM stock) corresponding to the non-mammalian miRNA; cel-miR-39 (5′-UCACCGGGUGUAAAUCAGCUUG-3ʹ, IDT, Leuven, Belgium), was added to the TRIzol LS/liquid mixture to enable miRNA normalization between samples collected at the different time points [49]. RNA was extracted after collection of all samples.

RT-qPCR studies were designed to conform to community standards (the MIQE guidelines [63]) where appropriate or possible. Our methods for miRNA detection and quantification have previously been described in detail [49,64]. Briefly, cDNA synthesis was performed using the MicroRNA Reverse Transcription Kit (Life Technologies) and appropriate miRNA-specific hairpin RT primers in multiplex format. Subsequently, miRNAs were amplified using Small RNA TaqMan assays and TaqMan Gene Expression Master Mix on a Step-One Real-Time PCR instrument (all Thermo Fisher Scientific) as according to manufacturer’s instructions. miRNA assay product IDs are shown in Table S1. Universal cycling conditions were used; 10 minutes at 95ºC, followed by 40 cycles of 95ºC for 10 seconds and 60ºC for 1 minute.

For absolute quantification, sample miRNA quantities were compared to a 10-fold dilution series of synthetic miRNA oligonucleotides (IDT) that were reverse transcribed and amplified in parallel to experimental samples. Absolute quantification of the external spike was used to correct for differences in extraction efficiencies between samples. The cel-miR-39 copies per sample were divided by the mean of all cel-miR-39 measurements to generate a scale factor (such that the mean of all scale factors was equal to one). miRNA-of-interest copies where then divided by the scale factor for each sample in order to normalize the data while preserving the natural range of copy number values. To obtain serum miRNA concentrations (copy numbers per milliliter biofluid), the ratio of input volume used for extraction, resuspension of RNA, reverse transcription and PCR was calculated and the normalized miRNA copy number values scaled accordingly. We have previously observed that all miRNA TaqMan assays are linear down to 300 copies per PCR reaction (although lower concentrations were not tested) [14]. For the purposes of this study we defined the LOQ as this value (i.e. 300) multiplied by the same scaling factor described above (to convert the LOQ to copies per ml of biofluid).

Relative quantification was performed using the Pfaffl method [65]. PCR reaction efficiencies were determined empirically using LinRegPCR [66].

### EV isolation

C2C12 cells were seeded in ten 150 mm plates in GM, medium was switched to DM for six days to induce myogenic differentiation and conditioned medium was collected (two days after the last medium change). Extracellular vesicles were isolated from differentiated C2C12 myotube conditioned medium by size-exclusion liquid chromatography (SEC-LC) as described previously [52]. Briefly, debris from the crude conditioned medium samples was removed by centrifugation at 300 *g* for five minutes, followed by 2,000 *g* for ten minutes in fresh tubes. Samples were passed through 0.22 µm filters and then run through a 10 kDa tangential flow membrane to concentrate the sample to ~15 ml volume, followed by a further filtration step using 10 kDa spin filters (Sartorius, Goettingen, Germany) (centrifugation at 4,000 *g* for 15 minutes at 4°C) to further concentrate the sample to a volume less than 2 ml. Concentrated samples were injected into a Sephacryl column containing Sepharose 4 Fast Flow resin (GE Healthcare, Little Chalfont, UK) and linked to an ÄKTA pure system with UV flow cell (GE Healthcare) for size-exclusion fractionation. Fractions were collected based on UV absorbance (protein content) and the EV-containing fractions (numbers five to eight) were pooled and further concentrated to a volume of ~200 µl using a 10 kDa spin filter (MilliporeSigma).

### Nanoparticle tracking analysis

Nanoparticle tracking analysis was performed using the NanoSight NS500 instrument and NTA 3.1 analytical software (both Malvern Instruments, Malvern, UK). Samples were diluted 1:500 in PBS to achieve a particle count of between 2 × 10^8^ and 2 × 10^9^ particles per ml. Three 30 second videos for each sample were recorded and analyzed using the script and batch control facilities.

### Western blot

EV sample total protein concentrations were determined by BCA assay (Thermo Fisher Scientific, Loughborough, UK), and 10 µg of protein separated by SDS-PAGE using NuPAGE Sample Reducing Agent and precast NuPAGE 4–12% Bis-Tris Protein Gels (both Thermo Fisher Scientific). Precision Plus Protein Dual Color Standards ladder was utilized to determine the size of protein bands (Bio-Rad Laboratories, Perth, UK). Proteins were transferred to a polyvinylidene difluoride (PVDF) membrane and probed with antibodies as appropriate (details of primary and secondary antibodies are provided in Table S2). Chemiluminescence was measured using the Odyssey Fc Imaging System (LI-COR Biosciences, Cambridge, USA).

### Statistical analysis

Correlation analysis, linear regression, and non-linear regression were performed using GraphPad Prism 5 (GraphPad Software Inc, La Jolla, CA).
