# Supplementary material for: Extracellular microRNAs exhibit sequence-dependent stability and cellular release kinetics
Source: RNA Biol. 2019 Mar 5;16(5):696–706. doi: 10.1080/15476286.2019.1582956 (PMC6546368; doi:10.1080/15476286.2019.1582956)
Supplement: Supplemental Material [file krnb-16-05-1582956-s001.docx]

| **miRNA** | **Assay ID** | **Target Sequence** |
| --- | --- | --- |
| **miR-1a-3p** | 002222 | UGGAAUGUAAAGAAGUAUGUAU |
| **miR-16-5p** | 000391 | UAGCAGCACGUAAAUAUUGGCG |
| **miR-133a-3p** | 002246 | UUUGGUCCCCUUCAACCAGCUG |
| **miR-206-3p** | 000510 | UGGAAUGUAAGGAAGUGUGUGG |
| **miR-223-3p** | 002295 | UGUCAGUUUGUCAAAUACCCCA |
| **let-7a-5p** | 000377 | UGAGGUAGUAGGUUGUAUAGUU |
| **cel-miR-39** | 000200 | UGUCAGUUUGUCAAAUACCCCA |

**Table S1**

**List of RT-qPCR assays used in this study.**

Product IDs for Small RNA TaqMan RT-qPCR assays used in this study (all Thermo Fisher Scientific).

| **Primary Antibody** | **Host** | **ID** | **Manufacturer** | **Dilution** | **Expected Size** |
| --- | --- | --- | --- | --- | --- |
| Anti-ALIX | Mouse mAb | Ab117600 | AbCam | 1:1,000 | 96 kDa |
| Anti-TSG101 | Rabbit mAb | Ab125011 | AbCam | 1:5,000 | 45 kDa |
| Anti-CD81 | Rabbit mAb | Ab109201 | AbCam | 1:1,000 | 26 kDa |
|  |  |  |  |  |  |
| **Secondary Antibody** |  |  |  |  |  |
| Anti-Mouse IgG-HRP | Goat | 7074 | CST | 1:5,000 |  |
| Anti-Rabbit IgG-HRP | Goat | 7076 | CST | 1:5,000 |  |

**Table S2**

**List of antibodies used in this study.**


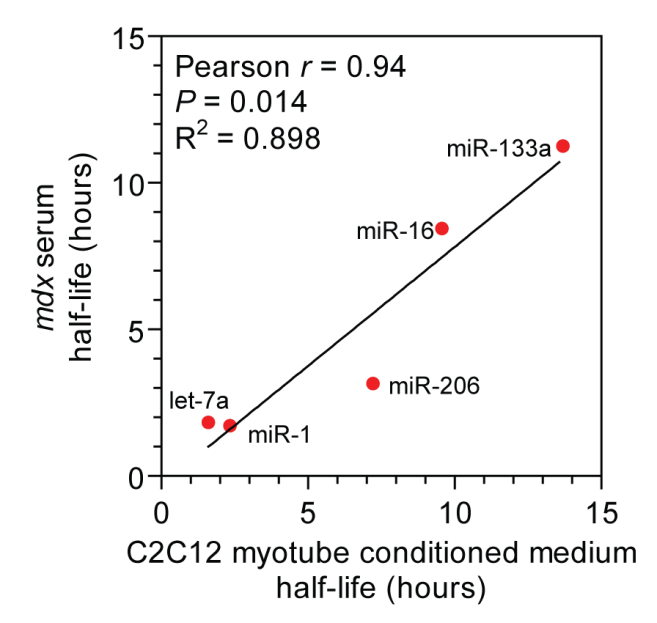


**Figure S1**

**Correlation of miRNA half-lives in C2C12 conditioned medium and dystrophic serum.**

**
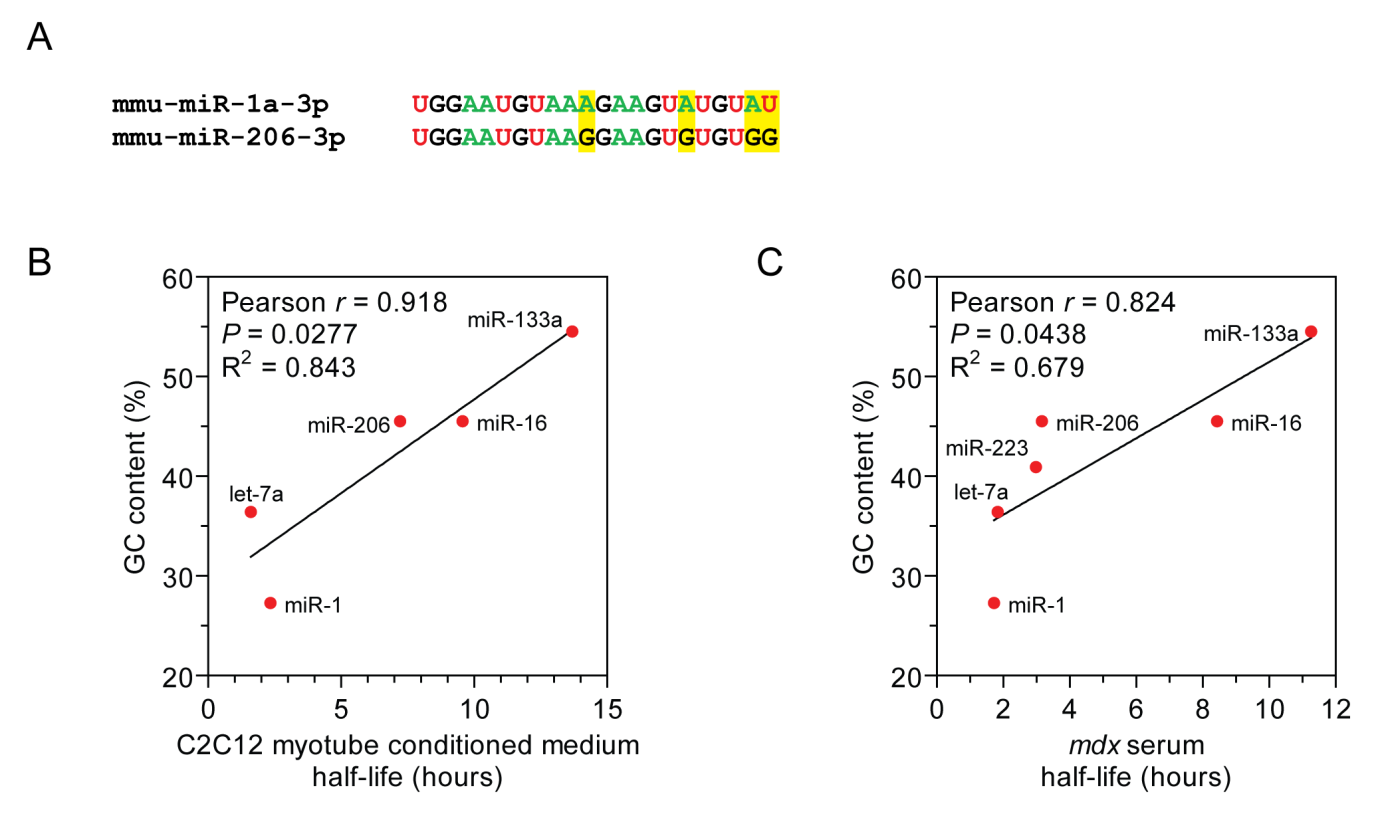
**

**Figure S2**

**Extracellular miRNA stability is a correlated with GC content.**

(A) Sequences of miR-1 and miR-206. Differences between the sequences are highlighted in yellow. Correlation between ex-miRNA half-life and GC content for (B) C2C12 myotube conditioned medium, and (C) serum, respectively.


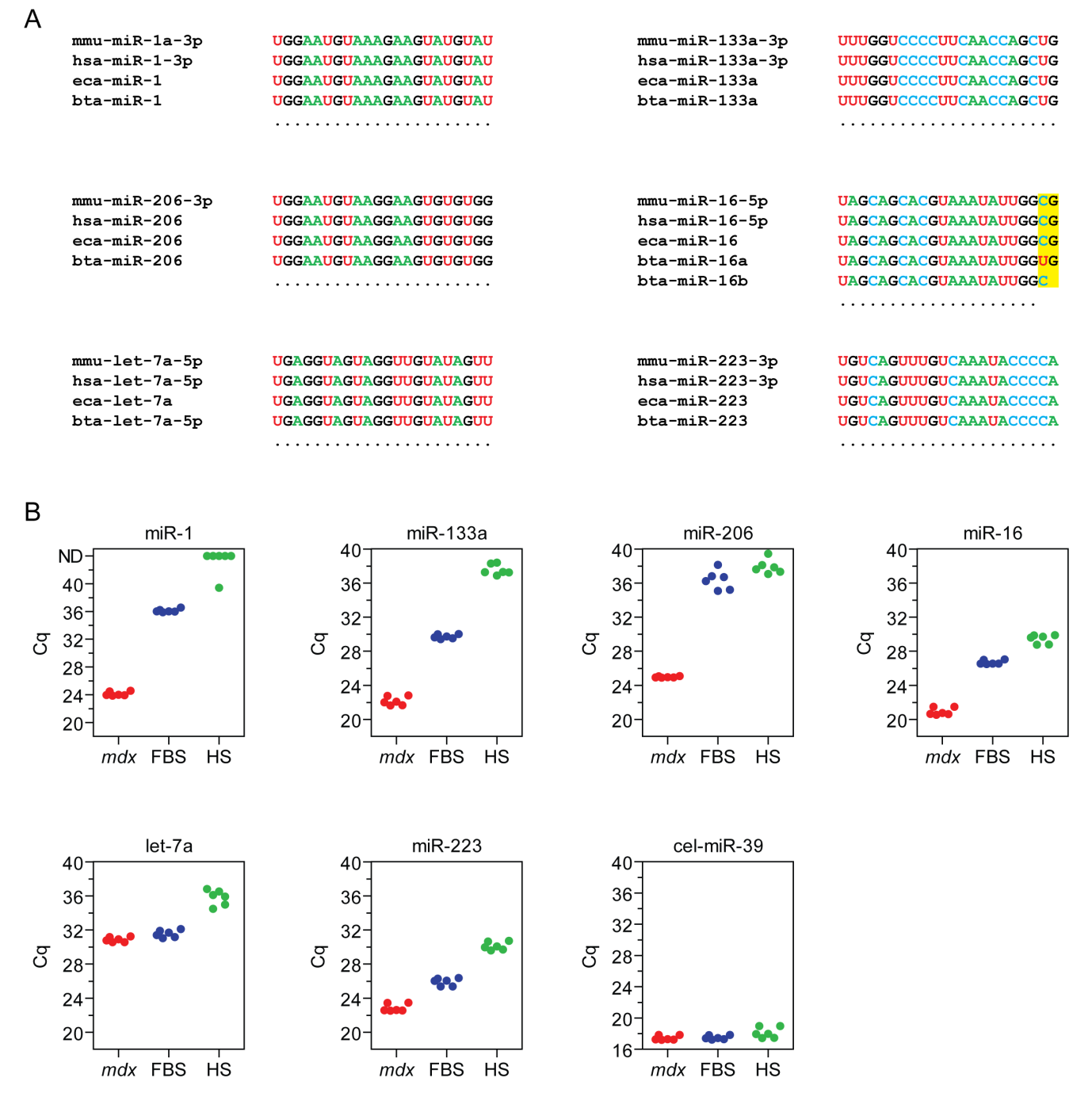


**Figure S3**

**Measurement of extracellular miRNAs in cell culture serum additives.**

(A) Alignment of mature miRNA sequences between species; *Mus musculus* (mmu), *Homo sapiens* (hsa), *Bos taurus* (bta), and *Equus caballus* (eca). Differences between the sequences are highlighted in yellow. miRNA sequences were retrieved from miRBase v22 in May 2018. (B) Background miRNA levels were measured in undiluted fetal bovine serum (FBS) and horse serum (HS), and compared to *mdx* serum which served as a positive control. The non-mammalian miRNA, cel-miR-39 (from *Caenorhabditis elegans*), was added as a synthetic oligonucleotide at the phenol extraction phase and served as an external spike-in control in order to directly compare between samples. ND, not detected.
